# Engineering MSC Migration: Roles of Nanoparticles in Activating Migratory Pathways and Functions

**DOI:** 10.3390/ijms27062530

**Published:** 2026-03-10

**Authors:** Temuulen Batsaikhan, Hyun Su Lee, Young Joon Seo

**Affiliations:** 1 Department of Otorhinolaryngology, Wonju College of Medicine Yonsei University, 20 Ilsan-ro, Wonju 26426, Republic of Korea; temuulen.ent@gmail.com (T.B.); glims1203@gmail.com (H.S.L.); 2 Research Institute of Hearing Enhancement, Wonju College of Medicine Yonsei University, 20 Ilsan-ro, Wonju 26426, Republic of Korea; 3 Department of Convergence Medicine, Wonju College of Medicine Yonsei University, 20 Ilsan-ro, Wonju 26426, Republic of Korea

**Keywords:** nanoparticle, mesenchymal stem cell, homing, cell tracking, nanomedicine

## Abstract

Mesenchymal stem cells (MSCs) hold strong therapeutic potential due to their regenerative, anti-inflammatory, and immunomodulatory properties. A key factor in their effectiveness is the ability to home in to injured sites. However, clinical outcomes are limited by poor homing efficiency, insufficient migration, tracking challenges, and risks of unwanted differentiation. This review explores the molecular mechanisms of MSC homing, particularly the CXCR4/SDF-1 axis and matrix remodeling. We highlight recent advances in using nanoparticles—such as magnetic, silica, and polymer-based systems—to enhance chemokine receptor expression and homing. Future directions include MSC engineering, advanced tracking, and AI-guided delivery strategies to improve therapeutic efficacy.

## 1. Introduction

Stem cells comprise diverse populations with distinct self-renewal and differentiation capacities depending on their tissue of origin and developmental stage. These biological properties have driven extensive research across multiple fields due to their therapeutic potential. Mesenchymal stem/stromal cells (MSCs) are particularly attractive for cell therapy through their differentiation capacity, paracrine signaling, and immunomodulatory effects. However, effective clinical application requires not only functional differentiation but also the ability of transplanted cells to localize to the injured area. Importantly, migratory and niche-seeking behavior (homing) is not a universal feature of all stem cells. For example, hematopoietic stem/progenitor cells exhibit physiologically required trafficking between bone marrow and peripheral blood, whereas many tissue-resident stem cells primarily function within local niches and do not require systemic migration. Moreover, homing is not exclusive to stem cells; other cells including immune cells, progenitors, and tumor cells also exhibit specialized homing behavior.

Stem cells are categorized based on their origins. Among these, mesenchymal stem cells (MSCs) are widely used in cell-based therapies due to their accessibility from adult or perinatal tissues and reduced ethical concerns compared with embryonic stem cells; they exhibit lower risk of tumorigenicity relative to pluripotent stem cells, while retaining high proliferative capacity and self-renewal potential. In clinical and translational research, MSCs have been widely studied and applied in the treatment of cancer [1], regenerative therapy [2], injury [3], and inflammation [4]. These conditions represent a substantial global health burden; for example, cancers accounted for approximately 250 million disability-adjusted life years (DALYs) worldwide in 2019 [5], while musculoskeletal disorders contributed more than 150 million DALYs, highlighting the urgent need for improved regenerative and therapeutic strategies [6]. In spinal cord injury, clinical studies have reported significant improvements in sensory outcomes following MSC therapy compared with placebo controls [7,8]. Johnson et al. (2021) conducted a systematic review and meta-analysis (19 human and 28 animal) that identified significant functional improvements following MSC transplantation in both human and animal studies; however, differences in transplantation timing, MSC source, and outcome assessment across species may limit the predictive value of animal models and challenge clinical translation [9].

Yunlong Shan et al. reviewed the fate of MSCs following transplantation and summarized a variety of biological responses in transplanted MSCs, including apoptosis, autophagy, differentiation, ferroptosis, phagocytosis, and senescence. These cellular fates are influenced by the local tissue microenvironment encountered after transplantation, including factors such as hypoxia, inflammatory cytokines, immune cell interactions, and oxidative stress, as well as by the route of MSC administration, which determines biodistribution and exposure to systemic versus site-specific signals. The persistence of transplanted MSCs in vivo remains controversial; while some studies resulted in rapid clearance within days [10], others showed persistence of viable cells up to 120 days [11]. Short-term survival may lead to insufficient therapeutic efficacy, whereas prolonged persistence raises safety concerns, including for tumorigenesis or abnormal differentiation [12].

In systemic administration, MSCs are delivered intravenously, primarily to exert paracrine and immunomodulatory effects. In this context, therapeutic efficacy depends on effective trafficking from circulation to target areas. However, it is limited by the low proportion of MSCs that successfully reach target sites, as a substantial fraction become sequestered in non-target organs, particularly the lungs, resulting in a pulmonary first-pass effect [13,14]. This sequestration may induce oxidative stress and inflammatory response, potentially leading to apoptosis or senescence and thereby limiting both target-site engraftment and therapeutic effect. This limited biodistribution has been described as a “hit and run” mechanism [15].

In contrast, local administration involves direct delivery of MSCs into or near the injured areas to promote regeneration. In this setting, therapeutic outcomes depend on cell retention, survival, and integration within the injured tissue [16]. Accordingly, reduced clinical efficacy may result from inadequate systemic homing or insufficient local retention, depending on the delivery strategy. Despite strong therapeutic potential, only a small fraction of administered MSCs successfully migrate to and engraft within target tissues, leading to inconsistent treatment outcomes. Moreover, a limited ability to track MSC biodistribution, viability, and function in vivo further hampers clinical translation, despite promising preclinical results [17,18].

In this review, we summarize the molecular mechanisms of MSC homing, focusing on chemokine- and integrin-mediated signaling pathways, and examine recent advances in nanoparticle-based strategies to enhance MSC migration, targeting, and in vivo tracking. Furthermore, we also review current experimental and clinical challenges, along with safety concerns related to in vitro expansion and nanoparticle labeling. We then outline future directions, including molecular engineering, advanced imaging, and data-driven approaches, that may improve therapeutic outcomes and the translational potential of MSC-based therapies.

## 2. Mechanisms of Homing

**Figure 1:**
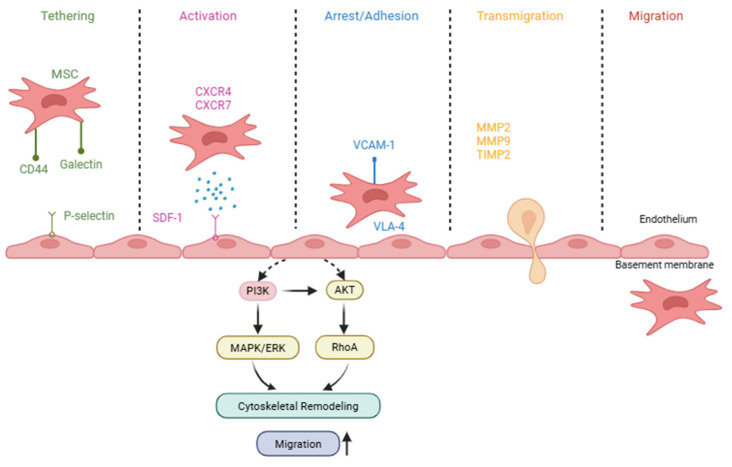
Mechanisms of Mesenchymal Stem Cell (MSC) Homing Cascade. Schematic illustration of the MSC homing cascade, including tethering, chemokine-mediated activation (SDF-1/CXCR4), integrin-dependent adhesion (VLA-4/VCAM-1), transmigration through the endothelium, and migration regulated by signaling pathways such as PI3K/AKT and MAPK/ERK.

One of the key reasons for the widespread applications of MSC in research is their intrinsic homing ability—the capacity to migrate towards injury sites, inflammation and disease. In this review, the term ‘homing’ refers specifically to the active trafficking of MSCs from the circulation to target tissues via two ways. Importantly, this biological property is distinct from the method of cell administration. In local homing, MSCs are directly delivered into or near the target area, where they respond to local chemokine gradients. In systemic homing, MSCs, whether administered or endogenous, enter the bloodstream and migrate to target tissues through a multistep process. These steps consist of: (1) tethering and rolling along the vascular endothelium, (2) activation by chemokine and cytokines, (3) arrest through integrin-mediated adhesion, (4) transmigration across the endothelial barrier, and (5) migration through the extracellular matrix [19,20]. The multistep process of MSC homing, including endothelial tethering, chemokine-mediated activation, integrin-dependent adhesion, and transmigration, is illustrated in Figure 1. While chemokine–receptor interactions initiate migration, effective homing requires coordinated activation of downstream molecular cascades that enable MSCs to respond dynamically to injury sites.

During the initial tethering and rolling step, Nitzsche F. et al. [21] compared leukocytes and MSCs, highlighting a key difference: unlike leukocytes, MSCs do not express selectin receptors. However, another study reported that MSCs can adhere to endothelial cells in a P-selectin dependent manner, despite lacking known P-selectin ligands, suggesting the involvement of other ligands [22].

Following this step, chemokine receptor family—particularly the CXCR4/SDF-1 axis—is implicated as a contributing mechanism in MSC homing. Stromal cell-derived factor 1 (SDF-1, also known as CXCL12), which is secreted from injury sites, binds to CXCR4 receptors on the MSC surface, directing their migration toward the site of injury. In addition, this axis also interacts mechanotransduction pathways that regulate cytoskeletal dynamics and cell–matrix adhesion, enabling MSCs to adapt to variations in extracellular matrix stiffness during migration and tissue infiltration [23]. Recent studies have emphasized the importance of maintaining or enhancing CXCR4 expression to improve homing efficiency [24,25]. Binding of SDF-1 to its receptor CXCR-4, a G-protein coupled receptor, activates heterotrimeric G proteins that initiate intracellular signaling cascades essential for cell migration. Among the downstream pathways activated is the phosphatidylinositol 3-kinase (PI3K/Akt) signaling pathway. For example, Ling et al. (2022) showed that human amnion-derived MSC expressing CXCR4 exhibited SDF-1 induced migration, possibility of activation of PI3K/Akt signaling pathway, in a chemotherapy-induced premature ovarian insufficiency model [26]. Similarly, in liver injury model, the therapeutic effects of lentiviral-mediated CXCR4 overexpression of transplanted bone marrow-derived mesenchymal stem cells (BMSC) were shown to be dependent on PI3K/Akt pathway activation. Pharmacological inhibition of PI3K/Akt signaling significantly reduced the number of homed transplanted BMSCs, and mRNA expression levels of SDF/CXCR-4 were significantly decreased [27]. In parallel, the mitogen-activated protein kinase/extracellular signal-regulated kinases 1/2 (MAPK/ERK1/2) pathway is also activated via the CXCR4/SDF-1 axis. Li et al. (2018) [28] confirmed that SDF-1 binding to CXCR4 in MSCs induces activation of both focal adhesion kinase (FAK) and ERK signaling, leading to enhanced cytoskeletal reorganization and cell migration. This chemotactic response is mediated through MAPK signaling cascades that converge on ERK1/2 activation [28]. In addition to the PI3K/Akt and MAPK/ERK1/2 pathways, Janus kinase/signal transducer and activator of transcription (JAK/STAT) is involved during inflammatory conditions. Long-term exposure of MSCs to tumor cell-conditioned medium has been shown to activate the JAK/STAT pathway [29], which regulates key cellular processes including cell proliferation, differentiation, apoptosis, and cell migration in multiple cell types. In a hypoxic tumor model, Jiang X et al. (2018) demonstrated that polymeric nanoparticles induced CXCR4 overexpression in human adipose-derived stem cells, enhancing their migration into the tumor core [30]. Also, Teo J.Y et al. (2020) reported that surface modification of MSC with SDF-1-loaded nanoparticles significantly improved chemotactic response and homing to ischemic tissue [31]. Collectively, these studies indicate that the CXCL12/SDF-1–CXCR4 axis is an important but insufficient determinant of MSC recruitment; native MSCs often express CXCR4 at low levels, and effective homing typically requires coordinated regulation of chemokine responsiveness together with adhesion molecules, cytoskeletal signaling, and matrix remodeling.

In the next step, firm adhesion or arrest is mediated by integrin. Integrin-mediated firm adhesion contributes to MSC arrest on activated endothelium. MSCs express multiple integrins and adhesion molecules, including α1, α2, α3, α4, α5, β1, β3, as well as VCAM-1 ICAm-1, ICAM-3, and CD166, whereas β2 is not expressed on MSC’s surface [32]. Under shear flow conditions, neutralization of VLA-4 significantly reduces firm adhesion of hMSCs to endothelial cells (Brigitte et al., 2006) [22]. Similarly, Steingen et al. (2008) reported that blocking VCAM-1 and VLA-4 significantly reduced MSC adhesion in HUVEC monolayer, indicating that integrin-mediated interaction are required for efficient MSC transendothelial migration [33]. Activation of the chemokine receptor further induces intracellular signaling through small Rho family GTPases, which regulate key cellular processes including migration, adhesion, transcription, growth, differentiation, and vesicular trafficking. Among these proteins, RhoA plays a critical role in regulating actin and microtubule dynamics, and is believed to be essential for effective MSC migration. Under hypoxic condition, MSCs exhibit enhanced migratory capacity accompanied by increased RhoA activation, suggesting that Rho signaling contributes to improved homing efficiency [34,35].

The forth step is transmigration; in this step MSC should move across the endothelial layer and basement membrane. This process is facilitated by matrix metalloproteinases (MMPs), which helps to degrade extracellular matrix components. Song et al. (2011) [36] reported that CXCR4 expression in MSCs is upregulated following exposure to tumor-conditioned medium, and pharmacologic inhibition of CXCR4 partially reduced MSC migration in vitro and tumor homing in vivo. Importantly, inhibition of MMP-2 also impaired migration, suggesting that chemokine signaling cooperates with matrix remodeling during MSC recruitment. These findings indicate that CXCR4 and MMP-2 contribute to MSC migration but are not solely sufficient to drive effective homing [36]. However, the exact interactions of MSC transmigration remain incompletely understood.

Autophagy has also been reported to modulate MSC migratory and immunoregulatory behavior by influencing cellular stress resistance and secretome composition; for example, autophagy-related changes can alter chemokine/cytokine secretion, which indirectly shapes immune cell trafficking and local inflammatory gradients. Cen et al. (2019) [37] reported that autophagy enhances MSC-mediated CD4+ T cell migration and immunomodulation through increased secretion of CXCL8 and TGF-β1. However, this is not the direct result of migration of MSCs themselves; rather, MSC autophagy can facilitate the migration and anti-inflammatory activity of surrounding immune cells [37].

## 3. Enhancement of MSC Homing Using Nanoparticles

**Figure 2:**
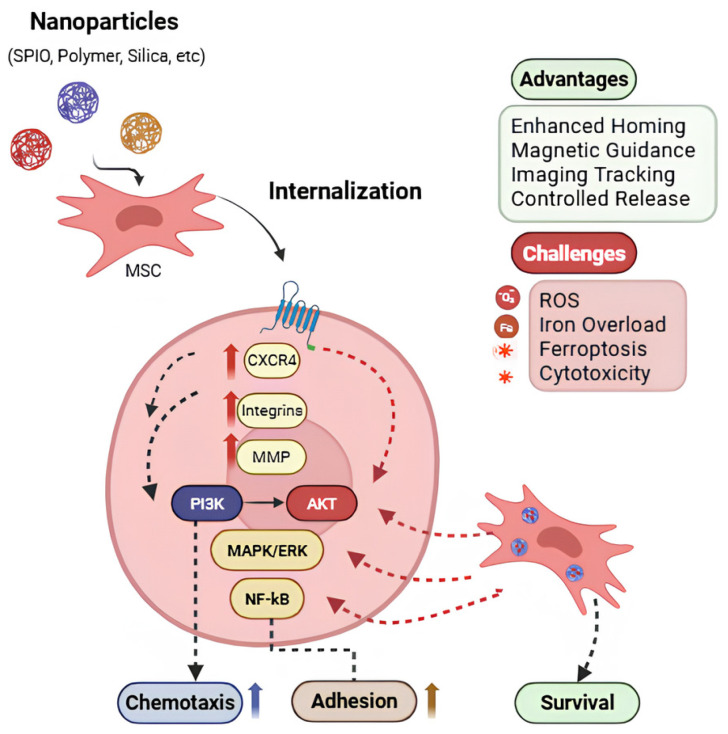
Schematic representation of nanoparticle-enhanced MSC migration. Nanoparticles modulate chemokine receptor expression and intracellular signaling pathways, thereby improving MSC homing, targeting efficiency, and in vivo tracking.

Given the multifactorial regulation of MSC homing, various strategies, including nanoparticle-based approaches, have been developed to enhance their targeted migration. These include magnetic nanoparticle-based targeting, nanoparticle-induced enhancement of migratory capacity, and upregulation of key homing-related chemokines and cytokines. Together, these approaches aim to improve MSC localization and therapeutic efficacy at sites of injury or disease. The role of nanoparticles in modulating MSC migratory pathways and homing efficiency is summarized in Figure 2.

Understanding nanoparticle–cell interactions are important for elucidating their effects on MSC homing, migration, differentiation and regeneration. While numerous studies have explored these interactions, the precise underlying mechanisms remain incompletely understood. What is known thus far indicates that nanoparticle outcomes depend on type, shape, material, concentration and specific cell types. Key nanoparticle types, MSC sources, proposed mechanisms, and reported outcomes are summarized in Table 1.

Nanoparticles can be internalized by MSCs through several endocytic pathways, including clathrin-mediated endocytosis, caveolin-dependent uptake, or macrophinocytosis. During macropinocytosis, cells form large vesicles that engulf substantial volumes of extracellular fluid, allowing the internalization of micro-sized particles while potentially avoiding rapid degradation of vesicular contents. In clathrin-mediated endocytosis, ligands bind to specific plasma membrane surface receptors to form ligand–receptor complexes, which subsequently migrate to clathrin-rich sites and are internalized into the cell as coated vesicles. In contrast, caveolae-mediated endocytosis involves flask-shaped membrane invaginations composed primarily of the structural protein caveolin-1. Unlike clathrin-mediated and macropinocytic pathways, the caveolae-dependent pathway often bypasses lysosomal degradation, enabling prolonged intracellular retention of nanoparticles and sustained signaling effects [44,45].

Several studies have demonstrated that nanoparticle internalization can directly enhance MSC homing-related signaling and migratory behavior.

Iron-containing nanoparticles:

Iron oxide-based nanoparticles are among the most extensively studied platforms. In studies, iron oxide nanoparticles showed enhanced MSC migration along with increased CXCR4 expression in response to SDF-1α at target sites [46,47]. Liu et al. (2023) showed superparamagnetic iron oxide particles (SPIOs) upregulated chemokine receptor gene expression, including CXCR4, CCR5, CCR10, CXCR3, CXCR5, and CXCR7; however, they did not enhance MSC migration in vitro, indicating that receptor expression alone was insufficient without corresponding chemokine gradients [38]. Supporting this concept, another study demonstrated that overexpressed CXCR4 in MSC with nanoparticles induced MMP expression, including MMP 9 and MMP2, via activation of PI3K/Akt/NF-kB signaling pathways; pharmacological inhibitors of these pathways significantly reduced MSC migration, indicating that coordinated downstream signaling is required beyond receptor upregulation alone [48]. Moreover, PDA-coated Fe_3_O_4_ superparticles increased activation of CXCR4, CCR1, and c-Met in bone marrow-derived MSCs, further supporting the role of nanoparticle mediation of chemokine responsiveness [49].

Silica and polymer-based nanoparticles:

Silica (SiO_2_) and polymeric nanoparticles have been shown to increase homing ability. Vitale et al. (2022) reported that internalization of SiO_2_ NPs in human MSCs led to inhibition of autophagic flux, resulting in increased CXCR4 expression [39]. This upregulation improved MSC chemotaxis toward SDF-1α, and under hypoxic conditions mimicking tissue injury, where SDF-1α release further activated the CXCR4/SDF-1 axis to promote migration.

Titanium nanoparticles and cytoskeletal modulation:

Hou et al. (2013) reported that titanium nanoparticles could enhance MSC migration by altering cellular adhesion and cytoskeletal dynamics, with effects vastly dependent on particle size [50]. These findings highlight that nanoparticle-induced cytoskeletal remodeling represents an additional mechanism by which MSC migratory behavior can be enhanced.

Physical guidance strategies using magnetic nanoparticles:

Beyond biochemical modulation of homing pathways, nanoparticles can also enhance MSC targeting through physical guidance strategies. Dash et al. (2024) developed polymer-coated magnetic fluorescent nanoparticles that enhanced MSC migration and homing toward magnetic fields, and fluorescence provided tracking without affecting MSC viability or differentiation [40].

Collectively, these studies demonstrate that engineered nanoparticles can potentiate MSC homing by modulating receptor expression, intracellular signaling, and cytoskeletal organization. Although redox alterations were not specifically evaluated in the studies summarized in Table 2, nanoparticle internalization may influence intracellular oxidative balance through reactive oxygen species (ROS) generation, depending on particle composition and surface chemistry [51]. Reports of nanoparticle-driven modulation of chemokines/cytokines are summarized in Table 2.

In addition to homing enhancement, nanoparticles also serve as a tracking tool for MSCs. Nanoparticle-based labeling enables high-resolution, multimodal imaging of MSC biodistribution, persistence, and localization following transplantation. Peserico et al. (2022) highlighted that nanoparticle-based labeling enables high-resolution, multimodal tracking of MSCs in vivo, while also offering potential therapeutic and diagnostic functions through engineered surface modifications [52]. Importantly, separating homing enhancement mechanisms from tracking applications allows for clearer interpretation of nanoparticle effects on MSC behavior and facilitates the rational design of multifunctional platforms that balance targeting efficiency, imaging sensitivity, and cellular safety.

## 4. Methods to Study MSC Homing

MSC homing has been evaluated using a range of methods, from conventional in vitro migration assays to advanced nanoparticle-enabled in vivo tracking strategies. While classical methods such as wound healing (scratch assay) and transwell migration tests allow for the quantification of chemotactic responses toward gradients such as SDF-1α, these models lack tissue complexity and do not fully recapitulate physiological biodistribution.

**Figure 3:**
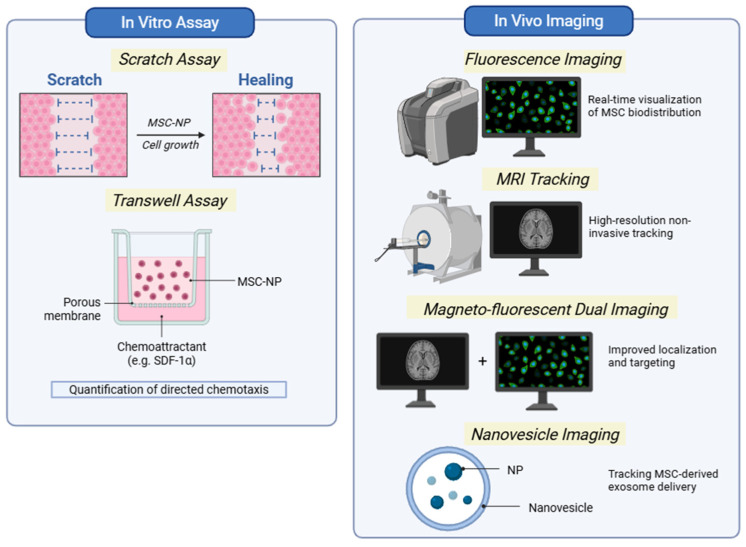
Methods to study MSC homing. Experimental approaches used to evaluate MSC homing, including in vitro migration assays (scratch and transwell assays) and in vivo imaging techniques such as fluorescence imaging, MRI tracking, dual magneto–fluorescent imaging, and nanovesicle-based tracking.

To address these limitations, nanoparticle-based labeling techniques have been increasingly adopted to enable real-time visualization and quantitative assessment of MSC homing in vivo. Magnetic nanoparticles (e.g., SPIOs), fluorescent nanoparticles, and dual-modal imaging platforms allow non-invasive tracking of MSC biodistribution, retention, and persistence using magnetic resonance imaging (MRI), fluorescence imaging, or combined modalities. These approaches provide improved spatial resolution and longitudinal monitoring compared to traditional histological analysis. Methods used to evaluate MSC migration and in vivo biodistribution are illustrated in Figure 3.

Importantly, nanoparticle-enabled tracking not only facilitates biodistribution analysis but also enables correlation between engineered homing enhancement strategies and actual in vivo targeting efficiency. The nanoparticle-based imaging and tracking methods discussed in this review are summarized in Table 3.

## 5. Preclinical Applications of Nanoparticle-Enhanced MSC Therapies

Various research has investigated MSC-based therapies across multiple fields, including injury, inflammation, cancer, tissue regeneration, and neurological disorders. While MSCs exhibit intrinsic therapeutic properties, their clinical efficacy is often limited by poor homing, insufficient retention at target area, and difficulty of monitoring cell fate in vivo. In recent years, various strategies—primarily evaluated in preclinical models—have been developed to enhance MSC migration, delivery, and tracking. The preclinical applications of nanoparticle-enhanced MSC therapies across different disease models are summarized in Table 4.

Among these approaches, magnetic nanomaterials have gained increasing attention due to their controllable magnetic properties and compatibility with biomedical applications. Magnetic nanoparticles enable diverse applications, including targeted drug delivery, magnetic hyperthermia therapy, imaging contrast enhancement, and biosensing. Advanced nanoscale characterization techniques, such as magnetic force microscopy (MFM), further allow investigation of magnetic interactions in biological systems at nanometer resolution [55].

In the context of MSC-based therapies, magnetic nanoparticle labeling has been reported to enhance migratory capacity and homing potential without significantly affecting cell viability, proliferation, or differentiation. Additionally, magnetic nanoparticles enable non-invasive MRI-based tracking of transplanted MSCs in vivo. Collectively, these properties highlight the potential of magnetic nanoparticle-engineered MSCs to improve targeting efficiency, monitoring, and therapeutic performance in stem cell-based therapies [56].

### 5.1. Tissue Regeneration and Musculoskeletal Disorders

In regenerative medicine, nanoparticle-enhanced MSC therapies have benefits in bone, cartilage, and muscle repair. Wang et al. (2022) reported that SDF-1α-loaded nanoparticles enhance the chemotactic effect of MSCs under both vitro and in vivo conditions, thereby promoting bone tissue regeneration [57]. Similarly, MSCs with steroid-loaded gold nanoparticles had strong anti-inflammatory effects, promoted cartilage regeneration, and suppressed ROS production [58]. These suggest that nanoparticle-mediated modulation of chemokine signaling and combination treatments may improve MSC-based musculoskeletal repair.

### 5.2. Neurological Disorders and Ischemic Injury

In neurological fields, conditions such as traumatic brain injury (TBI) and stroke neuro-inflammation and tissue necrosis contribute to neurodegenerative damage. Hai et al. (2017) developed an epidermal growth factor (EGF)-encapsulated MSC nanoparticle–collagen hydrogel and administered it in an ischemic injury model, resulting in nearly 100% blood perfusion at the targeted area without side effects [59]. Furthermore, in a TBI model, transplantation of human neural stem cells with curcumin-loaded nanoparticles showed significant improvement in brain edema, attenuation in astrogliosis, and decreased inflammatory markers [60].

### 5.3. Liver and Renal Diseases

In both acute and chronic liver injury murine models, zinc oxide nanoparticles significantly increased the homing ability of stem cells to injured liver tissue compared to healthy tissue in mouse, while also reducing inflammatory cytokine levels and improving tissue regeneration [41]. These findings highlight the dual effect of nanoparticles in improving MSC targeting and modulating inflammatory conditions. Similarly, in a diabetic nephropathy model, nanoparticles internalizing MSCs with an external magnetic field improved homing of cells to renal tissues and had anti-inflammatory and antifibrotic effects, suggesting the therapeutic potential of nanoparticle-assisted MSC delivery in renal diseases [61].

### 5.4. Cancer Therapy

Nanoparticle-assisted strategies have further improved the safety and efficacy of MSC-based cancer treatments by enhancing tumor targeting and controlling payload delivery. Butkiene et al. reported that nanoparticle-assisted photodynamic therapy (PDT) enhanced cancer treatment by improving the delivery, stability, and tumor targeting capabilities of MSCs without inducing toxicity, highlighting their potential as MSCs can be delivery vehicles for transportation [62]. However, careful consideration of tumor-promoting risks and long-term safety remains essential when applying MSC-based therapy in cancer.

### 5.5. Emerging Cell-Free and Tracking Applications

Beyond direct MSC transplantation, nanoparticle-induced MSC-derived exosomes have been studied. Iron oxide nanoparticle-labeled MSCs and nanovesicles increased levels of therapeutic molecules and reduced apoptosis and fibrosis, increasing angiogenesis and cardiac function recovery [42].

Furthermore, in liver and renal diseases, multifunctional nanoparticles have improved MSC therapy by facilitating precise homing, efficient cell labeling, and in vivo tracking through imaging techniques [63]. These advancements underline the potential of nanotechnology in stem cell-based regenerative medicine.

## 6. Challenges and Limitations

Even though numerous preclinical and experimental approaches have demonstrated positive outcomes and efficacy, there remain significant challenges and limitations in the transplantation of MSCs. A major limitation is the low proportion of MSCs that successfully migrate to injured tissues, reducing therapeutic efficacy. Additionally, systemically administered MSCs are frequently sequestered in non-target organs, particularly the lungs, where oxidative stress and inflammatory cytokines may induce apoptosis or senescence [20].

To overcome poor homing efficiency, various strategies have been explored, including the use of nanoparticles, the enhancement of homing chemokines, and magnetic attraction to targeted areas. However, nanoparticle-labeled MSCs have been shown to induce toxicity, particularly with iron core nanoparticles, which can trigger ferroptosis. This process involves the accumulation of intracellular iron and promotes lipid peroxidation through suppression of glutathione peroxidase 4 (GPX4) [12]. Furthermore, nanoparticle-induced reactive oxygen species (ROS) accumulation may activate stress-responsive signaling pathways, such as p53-mediated cell cycle arrest or NF-κB-driven proinflammatory responses, potentially compromising MSC therapeutic function.

Nanoparticle properties—including size, surface charge, coating materials, and intracellular retention—significantly influence cellular uptake and downstream responses. Variability in labeling efficiency may introduce heterogeneity in MSC populations, resulting in inconsistent migration behavior and therapeutic outcomes. Dose-dependent effects further complicate optimization, as insufficient loading may fail to enhance homing, whereas excessive loading increases toxicity risk [56].

Long-term biodistribution and clearance also remain incompletely understood. The persistence of nanoparticle-labeled MSCs or residual nanoparticles in non-target organs such as the liver, spleen, or lungs raises concerns regarding chronic accumulation and delayed toxicity [64,65].

Collectively, these challenges highlight the need for more advanced strategies that carefully balance enhanced homing efficiency with long-term safety and sustained therapeutic activity.

## 7. Future Directions

As this review focuses on engineering MSC migration through nanoparticle-mediated activation of homing pathways, future research should prioritize refining the precision, safety, and controllability of migratory modulation while integrating broader biological and technological advances that influence homing efficiency and targeting performance.

- Elucidation of extravasation mechanism: While significant progress has been made in identifying chemokine and adhesion molecule interactions, the precise steps by which MSCs transmigrate across the endothelial barrier into the injured area are still poorly described. These processes often vary depending on the MSC source and external modulators such as nanoparticles and cytokines. Future studies should focus on molecular profiling to uncover the dynamics of MSC extravasation, including the role of endothelial junction proteins, such as VE-cadherin and claudins, as well as the role of perivascular cells and extracellular matrix remodeling during MSC transmigration. Advanced molecular profiling approaches, including time-resolved phosphoproteomics and live-cell imaging, may provide deeper insights into how MSCs coordinate adhesion, cytoskeletal remodeling, and matrix degradation in different tissue microenvironments.
- Application of single-cell and spatial omics technologies: MSC populations are highly heterogeneous, and only a subset of transplanted cells may possess robust homing and migratory capacity. The application of single-cell RNA sequencing (scRNA-seq), spatial transcriptomics, and single-cell proteomics would enable identification of MSC subpopulations with superior migratory phenotypes and distinct signaling signatures. Advances in gene editing and single-cell analysis open avenues for tailoring MSCs to individual patients or disease states. Modifying chemokine receptor expression or preconditioning MSCs with individualized cytokine environments may significantly enhance therapeutic outcomes and homing capacity.
- Molecular engineering for enhanced homing: Advances in gene-editing technologies, such as CRISPR/Cas9 and epigenetic modulation tools, offer opportunities to precisely regulate key molecules involved in MSC homing. Targeted upregulation of chemokine receptors (e.g., CXCR4, CCR2, or CXCR7), integrins, or cytoskeletal regulators may significantly improve migratory responses without compromising cell safety. In parallel, transient genetic or epigenetic modifications—rather than permanent alterations—may provide safer approaches to enhance homing while minimizing the risks of tumorigenicity or abnormal differentiation.
- Development of Advanced Nanoparticles: In contrast to cell-intrinsic engineering, future nanoparticle development should focus on optimizing extrinsic delivery platforms to improve MSC targeting, retention, and safety. Key priorities include designing nanoparticles with high biocompatibility and controlled biodegradability, engineering surface ligands for tissue- or endothelium-specific targeting, and enabling the controlled release of chemokines or supportive factors to sustain local gradients. Additionally, nanoparticle systems should be optimized to minimize oxidative stress, iron-induced ferroptosis, and off-target accumulation while maintaining stable imaging signals for long-term in vivo tracking. Such material-level innovations will be critical for integrating homing enhancement with imaging and therapeutic functions in a clinically translatable manner.
- Long-term and real-time cell tracking: Current tracking technologies are limited by signal dilution, toxicity, and difficulty of distinguishing between live and dead cells. Future tracking strategies should aim to combine molecular imaging with functional readouts, enabling real-time assessment of MSC viability, differentiation status, and therapeutic activity in vivo. The development of biodegradable, multimodal imaging probes and reporter systems will be critical for accurately evaluating long-term MSC fate and safety in clinical settings.
- Integration with AI analytics: The increasing complexity of MSC homing data necessitates advanced analytical approaches. Artificial intelligence (AI) and machine learning algorithms can be applied to large-scale datasets derived from imaging, omics, and preclinical studies to identify predictive patterns of MSC migration and therapeutic response. Computational modeling may further assist in optimizing nanoparticle design, dosing strategies, and delivery routes, ultimately enabling personalized and precision-guided MSC therapies.
- Cell-free approaches: Beyond direct stem cell transplantation, MSC-derived exosomes represent an important role in clinical application. Compared with live stem cells, MSC-derived exosomes exhibit low immunogenicity and minimal toxicity, and are free of ethical issues or tumorigenic potential. In addition, they are easier to store, handle, and standardize while retaining many of the therapeutic properties of their parental MSCs, including immunomodulatory, anti-inflammatory, and regenerative effects.

## 8. Conclusions

Mesenchymal stem cells exhibit significant therapeutic potential due to their regenerative, immunomodulatory, and paracrine characteristics. However, their effective clinical use is limited by poor homing efficiency, short-term in vivo circulation, and safety issues related to in vitro expansion and systemic administration. The evidences showing that MSC homing is a multistep, highly coordinated process that involves chemokine signaling, integrin-mediated adhesion, cytoskeletal remodeling, and extracellular matrix degradation. Among these mechanisms, the CXCR4/SDF-1 axis is important because it activates downstream signaling pathways such as PI3K/Akt, MAPK/ERK, and Rho GTPases, which together regulate migration, survival, and cytoskeletal dynamics. However, in vitro expansion has been shown to regularly reduce the expression of key homing receptors, resulting in reduced responsiveness of MSCs to injury-associated chemokine gradients in vivo.

Nanoparticle-based strategies have become valuable approaches to overcome these limitations through enhancing homing of mesenchymal stem cells, improving targeted delivery, and enabling in vivo tracking. Various nanoparticle types, including polymeric, silica, magnetic, and metal-based systems, have shown to modulate chemokine receptor expression, intracellular signaling pathways, and cytoskeletal organization, leading to improved homing and therapeutic outcomes. Additionally, nanoparticle labeling enables multimodal imaging approaches that provide insights into MSC biodistribution and post-transplantation status [66]. However, nanoparticle-assisted approaches also face challenges, including those related to cytotoxicity, oxidative stress, and ferroptosis, especially in iron-based nanoparticles.

Looking forward, advances in single-cell and spatial omics technologies, molecular engineering, and bioinformatics are expected to provide deeper insights into MSC heterogeneity and homing mechanisms. Integration of next-generation nanoparticle platforms with precise molecular engineering and artificial intelligence-guided analysis may enable personalized, efficient, and safer MSC-based therapies. Overall, while significant challenges remain, continued interdisciplinary efforts combining stem cell biology, nanotechnology, and systems-level analysis hold strong promise for unlocking the full therapeutic potential of MSCs in regenerative medicine.

## Figures and Tables

**Table 1:** Nanoparticle types, MSC sources, homing mechanism, and outcomes.

NP Type	NP Size	NP Composition	NP Zeta Potential	MSC Source	Mechanism	Outcome	Reference
Polymeric NP	~180 nm	Amino group-terminated PBAE	43.22 ± 2.33 mV	Human adipose-derived MSCs	CXCR4 overexpression	Increased migration to tumor	[30]
SDF-1α-loaded NP	182 ± 5 to 216 ± 8 nm	PLGA-b-HA	-	Mouse bone marrow MSCs	Sustained SDF-1α release increase CXCR4/SDF-1α interaction	Improved homing to ischemic muscle	[31]
SPIO NP	10–50 nm	superparamagnetic iron oxide particles	-	Rat bone marrow-derived MSCs	Upregulated CCR5, CCR10, CXCR3, CXCR4, CXCR5, CXCR7	Receptor upregulation but no improvement in migration (gradient-dependent)	[38]
Silica NP	50 ± 2 nm	SiO_2_ NP	-	Human bone marrow MSCs	CXCR4 increase	Increased chemotaxis to SDF-1α	[39]
Magnetic–fluorescent polymer-coated NP	50 nm	Ferric chloride, molasses, alginate	−18 mV in MQ and − 20 mV in PBS	Human adipose-derived MSCs	Magnetic guidance and CXCR4 modulation	Under magnetic field, increased homing	[40]
Zinc oxide NP	56.2 ± 1.7 nm	Zinc oxide, PEG2K-Ce6	1.05 ± 0.64 mV	MSCs	Homing improved	Liver regeneration and homing improved	[41]
Iron oxide nanovesicle	20–30 nm	Iron oxide	-	Human bone marrow MSCs	Paracrine effects improved	Cardiac repair improved, decreased apoptosis and fibrosis	[42]
NP–peptide dual modified	3 nm 50 nm	CuO	3.54 mV −18.87 mV	Mouse bone marrow MSCs	Dual engineering for homing and differentiation	Cartilage regeneration increased	[43]

**Table 2:** Chemokine and cytokine modulation by nanoparticles.

Nanoparticle Type	Chemokine/Cytokine	Effects	Mechanism	Reference
SPIO (iron oxide)	CCR5, CCR10, CXCR3, CXCR4, CXCR5, CXCR7	↑ Receptor expression	When gradient is present, increases migration	[38]
Polymeric NP	CXCR4	↑ Receptor gene expression	Hypoxia-upregulated SDF-1α leads to increase via CXCR4/SDF-1α axis	[30]
PDA-coated Fe_3_O_4_	CCR1, CXCR4, c-Met	↑ Chemokine receptors	Enhanced MSC chemotaxis	[49]
Silica NP	CXCR4	↑ Receptor gene expression	Autophagy flux inhibition leads to increased chemotaxis	[39]
Zinc Oxide NP	TNF-α, IL-6, IL-1β	↓ Pro-inflammatory cytokines	Immunomodulatory effect in liver injury, promotes liver regeneration	[41]
Iron-oxide nanovesicles	Apoptosis, fibrosis, angiogenic factors	Apoptosis/Fibrosis ↓, Angiogenesis ↑	Exosome-based paracrine signaling modulation	[42]

**Table 3:** Nanoparticle-based in vivo tracking and imaging strategies for MSC homing.

Method	Nanoparticle Type	Description	Applications	Limitations	Reference
Fluorescence Imaging	Fluorescent dyes/magnetic–fluorescent NPs	Real-time fluorescent tracking	Biodistribution study (lungs, liver, tumor)	Signal dilution	[53]
MRI (SPIO labeling)	SPIO NPs	High-resolution, non-invasive	Infarct, tumor, liver homing	High dose may affect viability	[38,54]
Magneto–fluorescent tracking	Polymer magnetic–fluorescent NPs	Dual modal	MSCs targeting and localization	Complex synthesis	[40]
Nanovesicle imaging	Iron oxide-loaded vesicle	Exosome tracking through NP signal	Exosome delivery	Limited spatial depth	[42]

**Table 4:** Summary of preclinical applications of nanoparticle-enhanced MSC therapies.

Disease Model	Nanoparticle Type	Size (nm)	MSC Source	Delivery Method	Key Quantitative Outcome	Proposed Mechanism
Traumatic Brain Injury	Curcumin-loaded noisome NPs	60–90 nm	hNS/PCs	Local injection, NPs orally	General locomotor activity ↑ Brain edema, astrogliosis ↓	Inflammatory response
Cartilage repair	Steroid-loaded AuNP	132–150 nm	MSC	Intra-articular	ROS ↓, cartilage thickness↑	Anti-inflammatory modulation
Ischemic injury	EGF-NP hydrogel	—	MSC	Local scaffold	~100% perfusion recovery	Growth factor release
Diabetic nephropathy	Magnetic NP	—	MSC	IV + magnetic field	renal retention ↑	Magnetic targeting
Cancer therapy	dNP-Ce6	—	BM-MSC	Culture	MSCs delivery of dNP-Ce6 ↑	Enhanced payload delivery

## Data Availability

No new data were created or analyzed in this study. Data sharing is not applicable to this article.

## Abbreviations

The following abbreviations are used in this manuscript:

CXCR	Chemokine receptor family
MAPK	Mitogen-activated protein kinase
mRNA	Messenger ribonucleic acid
PI3K/Akt	Phosphatidylinositol 3-kinase
STAT	Signal transducer and activator of transcription
EGF	Epidermal growth factor
ERK1/2	Extracellular signal-regulated kinases 1/2
FAK	Focal adhesion kinase
JAK	Janus kinase
MMPs	Matrix metalloproteinases
MSC	Mesenchymal stem cell
PDT	Photodynamic
ROS	Reactive oxygen species
SDF-1	Stromal cell derived factor 1
SPIO	Superparamagnetic iron oxide particles
TBI	Traumatic brain injury
TGF	Transforming growth factor
VLA-4	Very late antigen
AI	Artificial intelligence
NP	Nanoparticle
